# Supplementary material for: Computational-Based Discovery of the Anti-Cancer Activities of Pyrrole-Based Compounds Targeting the Colchicine-Binding Site of Tubulin
Source: Molecules. 2022 Apr 30;27(9):2873. doi: 10.3390/molecules27092873 (PMC9101527; doi:10.3390/molecules27092873)
Supplement: Supplementary file 1 [file molecules-27-02873-s001.zip › molecules-1688954-supplementary.pdf]

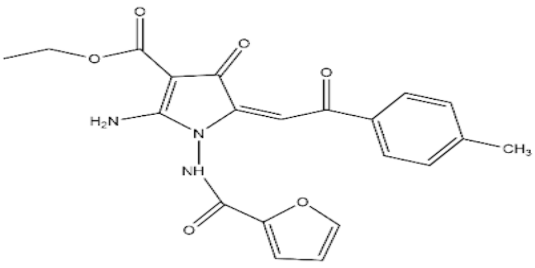

EAPC-64

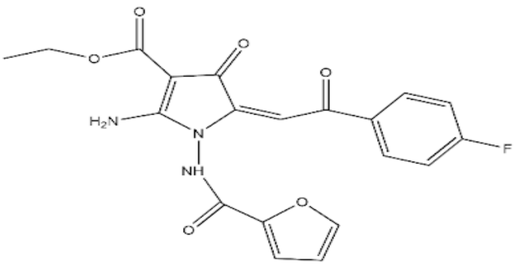

EAPC-69

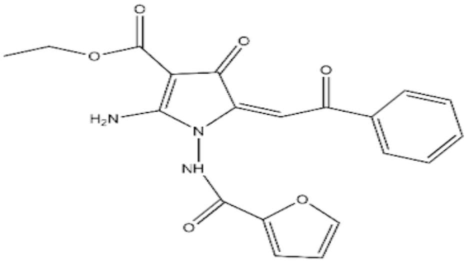

EAPC-65

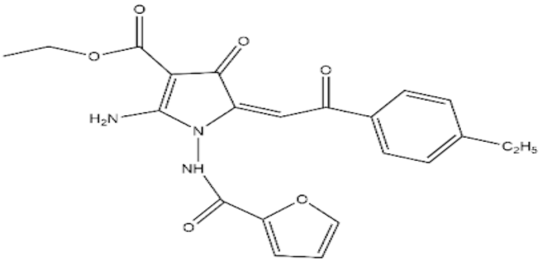

EAPC-70

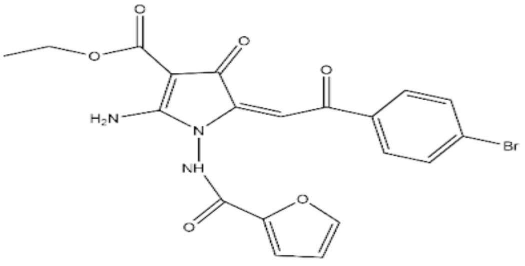

EAPC-66

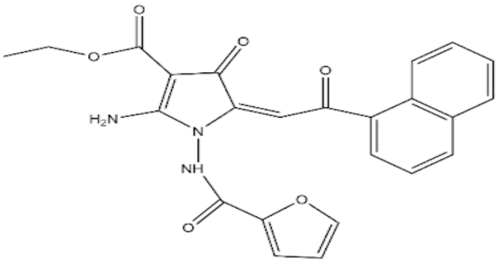

EAPC-71

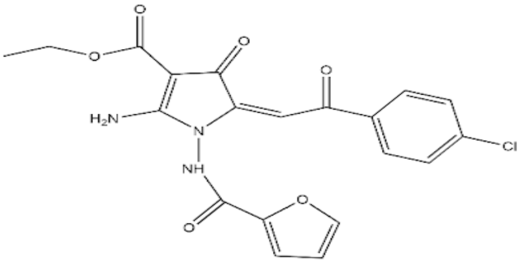

EAPC-67

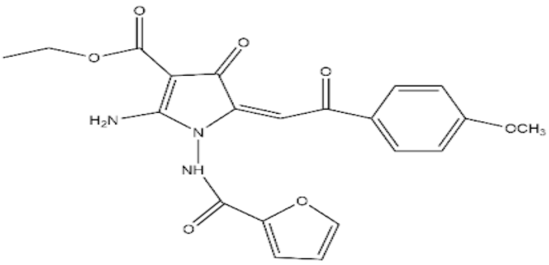

EAPC-72

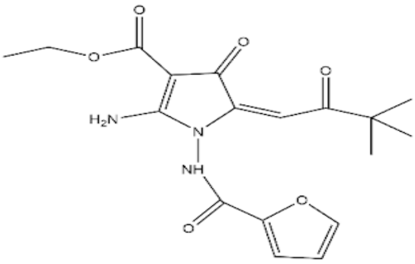

EAPC-68

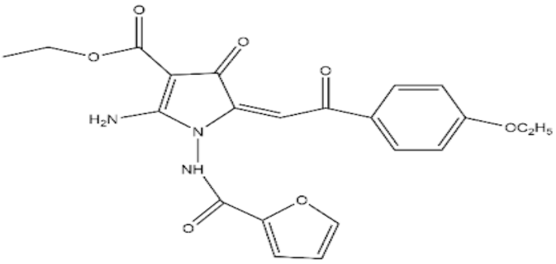

EAPC-73

Supplementary Figure S1. 2D structural formulas of synthesized EAPCs.

Supplementary Figure S2. Spectral characteristics of synthesized EAPCs

Ethyl (2-amino-5-(3,3-dimethyl-2-oxobutylidene)-1-(furan-2-carboxamido)-4-oxo-4,5-dihydro-1*H*-pyrrole-3-carboxylate (2a)

Yield 0.48 g (64%), yellow crystals, m. p. 258-259°C (ethanol). IR spectrum, n, cm^{-1}: 3369, 3258, 1704, 1682, 1655, 1590. ^{1}H NMR spectrum (DMSO–d_6), δ, ppm: 0.96 s (9H, t-Bu), 1.25 t (3H, Me, J 7.0 Hz), 4.18 q (2H, CH_2, J 7.1 Hz), 6.32 s (1H, CH), 6.68 double d (1H, furyl, J 3.5, 1.8), 7.27 double d (1H, furyl, J 3.5, 0.7), 7.94 double d (1H, furyl, J 1.8, 0.8), 8.38 br. s (1H, NH), 9.08 br. s (1H, NH), 10.59 br. s (1H, NH). ^{13}C NMR spectrum (DMSO-d_6), δ, ppm: 14.51, 26.15, 58.52, 84.80, 101.38, 111.84, 115.80, 140.55, 145.26, 146.09, 156.46, 163.62, 167.26, 174.26, 204.81. Found, %: C, 57.63; H, 5.61; N, 11.23. $C_{18}H_{21}N_3O_6$. Calculated, %: C, 57.59; H, 5.64; N, 11.19.

Ethyl 2-amino-1-(furan-2-carboxamido)-4-oxo-5-(2-oxo-2-phenylethylidene)-4,5-dihydro-1*H*-pyrrole-3-carboxylate (2b)

Yield 0.49 g (62%), yellow crystals, m. p. 240-241°C (ethanol). IR spectrum, n, cm_{-1}: 3354, 3330, 3121, 1694, 1660, 1627, 1608. ^{1}H NMR spectrum (DMSO–d_6), δ, ppm: 1.25 t (3H, Me, J 7.1 Hz) , 4.19 q (2H, CH_2, J 7.1 Hz), 6.64 s (1H, CH), 6.60 double d (1H, furyl, J 3.5, 1.5), 7.09 d (1H, furyl, J 3.4), 7.57 m ($5H_{arom.}$), 7.83 double d (1H, furyl, J 1.6, 0.6), 8.38 br. s (1H, NH), 9.07 br. s (1H, NH), 10.66 br. s (1H, NH). Found, %: C, 60.72; H, 4.36; N, 10.61. $C_{20}H_{17}N_3O_6$. Calculated, %: C, 60.76; H, 4.33; N, 10.63.

Ethyl 2-amino-1-(furan-2-carboxamido)-4-oxo-5-(2-oxo-2-(p-tolyl)ethylidene)-4,5-dihydro-1*H*-pyrrole-3-carboxylate (2c)

Yield 0.47 g (58%), yellow crystals, m. p. 253-254°C (ethanol). IR spectrum, n, cm^{-1}: 3331, 3264, 1703, 1685, 1659, 1621, 1593. ^{1}H NMR spectrum (DMSO–d_6), δ, ppm: 1.26 t (3H, Me, J 7.0 Hz) , 2.34 s (3H, Me), 4.21 q (2H, CH_2, J 7.1 Hz), 6.62 m (1H, CH, 1H, furyl), 7.10 d (1H, furyl, J 3.3), 7.25 d ($2H_{arom.}$, J 7.9 Hz), 7.64 d ($2H_{arom.}$, J 7.9 Hz), 7.85 double d (1H, furyl, J 1.6, 0.8), 8.43 br. s (1H, NH), 9.13 br. s (1H, NH), 10.76 s (1H, NH). Spectrum of NMR ^{13}C (DMSO-d_6), d, ppm: 14.46, 21.04, 58.47, 84.79, 102.08, 111.66, 115.49, 128.20, 129.06, 134.64, 145.73, 156.18, 163.61, 166.98, 174.05, 189.29. Found, %: C, 61.59; H, 4.71; N, 10.22. $C_{20}H_{17}N_3O_6$. Calculated, %: C, 61.61; H, 4.68; N, 10.26.

Ethyl 2-amino-5-(2-(4-ethylphenyl)-2-oxoethylidene)-1-(furan-2-carboxamido)-4-oxo-4,5-dihydro-1H-pyrrole-3-carboxylate (2d)

Yield 0.52 g (61%), yellow crystals, m. p. 249-250°C (ethanol). IR spectrum, n, cm^{-1}: 3409, 3201, 3119, 1709, 1646, 1627, 1608. ^{1}H NMR spectrum (DMSO–d_6), δ, ppm: 1.19 t (3H, Me, J 7.6 Hz) , 1.26 t (3H, Me, J 7.0 Hz), 2.64 q (2H, CH_2, J 7.6 Hz), 4.21 q (2H, CH_2, J 7.1 Hz), 6.62 dd (1H, furyl, J 3.5, 1.8), 6.64 s (1H, CH), 7.1 d (1H, furyl, J 3.1), 7.28 d ($2H_{arom.}$, J 8.3 Hz), 7.67 d ($2H_{arom.}$, J 8.3 Hz), 7.85 double d (1H, furyl, J 1.6 , 0.7), 8.41 br. s (1H, NH), 9.13 br. s (1H, NH), 10.76 s (1H, NH). Spectrum of NMR ^{13}C (DMSO-d_6), d, m.: 14.52, 14.98, 28.14, 58.55, 84.87, 102.42, 111.73, 115.55, 127.95, 128.38, 135.19, 141.00, 145.24, 148.80, 149.76, 156.68, 163.69, 167.05, 174.13, 189.56. Found, %: C, 62.14; H, 5.04; N, 9.89. $C_{22}H_{21}N_3O_6$. Calculated, %: C, 62.1; H, 5.00; N, 9.92.

Ethyl 2-amino-1-(furan-2-carboxamido)-5-(2-(4-methoxyphenyl)-2-oxoethylidene)-4-oxo-4,5-dihydro-1*H*-pyrrole-3-carboxylate (2e)

Yield 0.54 g (64%), yellow crystals, m. p. 240-241°C (ethanol). IR spectrum, n, cm^{-1}: 3364, 3331, 3127, 1709, 1657, 1621, 1608.

^1H NMR spectrum (DMSO–d_6), δ, ppm: 1.30 t (3H, Me, J 7.1 Hz), 3.85 s (3H, MeO), 4.24 q (2H, CH_2, J 7.1 Hz), 6.61 double d (1H, furyl, J 3.6, 1.8), 6.64 s (1H, CH), 6.97 d (2H, $H_{arom.}$, J 8.9 Hz), 7.1 double d (1H, furyl, J 3.5, 0.6), 7.74 d (2H, $H_{arom.}$, J 8.8 Hz), 7.82 double d (1H, furyl, J 1.7, 0.8), 8.27 br. s (1H, NH), 8.85 br. s (1H, NH), 10.47 s (1H, NH). Found, %: C, 59.32; H, 4.53; N, 9.86. $C_{21}H_{19}N_3O_7$. Calculated, %: C, 59.29; H, 4.50; N, 9.88.

Ethyl 2-amino-5-(2-(4-ethoxyphenyl)-2-oxoethylidene)-1-(furan-2-carboxamido)-4-oxo-4,5-dihydro-1*H*-pyrrole-3-carboxylate (2f)

Yield 0.62 g (70%), yellow crystals, mp. 230-231°C (ethanol). IR spectrum, n, cm^{-1}: 3333, 3133, 1712, 1654, 1622, 1605.

^1H NMR spectrum (DMSO–d_6), δ, ppm: 1.30 t (3H, Me, J 7.1 Hz), 1.37 t (3H, Me, J 6.9 Hz), 4.14 q (2H, CH_2, J 6.9 Hz), 4.21 q (2H, CH_2, J 7.1 Hz), 6.62 double d (1H, furyl, J 3.5, 1.8), 6.63 s (1H, CH), 6.95 d ($2H_{arom.}$, J 8.3 Hz), 7.10 double d (1H , furyl, J 3.4, 0.7), 7.72 d ($2H_{arom.}$, J 8.3 Hz), 7.82 double d (1H, furyl, J 1.6, 0.8), 8.27 br. s (1H, NH), 8.84 br. s (1H, NH), 10.47 s (1H, NH). Found, %: C, 60.15; H, 4.79; N, 9.59. $C_{22}H_{21}N_3O_7$. Calculated, %: C, 60.13; H, 4.82; N, 9.56.

Ethyl 2-amino-1-(furan-2-carboxamido)-5-(2-(naphthalen-1-yl)-2-oxoethylidene)-4-oxo-4,5-dihydro-1*H*-pyrrole-3-carboxylate (2g)

Yield 0.65 g (73%), yellow crystals, m. p. 272-273°C (ethanol). IR spectrum, n, cm^{-1}: 3370, 3260, 3241, 1700, 1677, 1655, 1590.

^1H NMR spectrum (DMSO–d_6), δ, ppm: 1.27 t (3H, Me, J 7.0 Hz), 4.21 q (2H, CH_2, J 7.1 Hz), 6.54 double d (1H, furyl, J 3.5, 1.7), 6.58 s (1H, CH), 7.11 double d (1H, furyl, J 3.5, 0.7), 7.53m ($3H_{arom.}$), 7.77 double d (1H, furyl, J 1.7, 0.7), 8.48 br. s (1H, NH), 9.20 br. s (1H, NH), 10.90 br. s (1H, NH). Spectrum of NMR ^{13}C (DMSO-d_6), d, ppm: 14.53, 58.59, 84.87, 104.62, 111.72, 115.71, 124.81, 125.09, 126.32, 127.61, 128.43, 129.31, 129.57, 132.87, 133.04, 135.39, 140.66, 145.19, 145.84, 156.72, 163.64, 167.19, 174.29, 192.58. Found, %: C, 64.75; H, 4.26; N, 9.46. $C_{24}H_{19}N_3O_6$. Calculated, %: C, 64.72; H, 4.30; N, 9.43.

Ethyl 2-amino-5-(2-(4-bromophenyl)-2-oxoethylidene)-1-(furan-2-carboxamido)-4-oxo-4,5-dihydro-1*H*-pyrrole-3-carboxylate (2h)

Yield 0.50 g (53%), yellow crystals, m. p. 275-276 °C (ethanol + dioxane). IR spectrum, n, cm^{-1}: 3414, 3336, 3125, 1709.1676, 1657, 1621.

^1H NMR spectrum (DMSO–d_6), δ, ppm: 1.25 t (3H, Me, J 7.1 Hz), 4.19 q (2H, CH_2, J 7.1 Hz), 6.57 s (1H, CH), 6.60 double d (1H, furyl, J 3.5, 1.8), 7.07 d (1H, furyl, J 3.4), 7.63 m ($4H_{arom.}$), 7.82 (1H, furyl), 8.34 br. s (1H, NH), 9.05 br. s (1H, NH), 10.68 br. s (1H, NH). Mass spectrum, m/z ($I_{rel.}$, %): 475 (97.7) [M]+, 473 (100.0) [M]+, 319 (51.1) [4-BrC6H4]+, 291 (12.7) [4- BrC6H4CO]+. Found, %: C, 50.68; H, 3.36; N, 8.89. $C_{20}H_{16}BrN_3O_6$. Calculated, %: C, 50.65; H, 3.40; N, 8.86.

Ethyl 2-amino-5-(2-(4-fluorophenyl)-2-oxoethylidene)-1-(furan-2-carboxamido)-4-oxo-4,5-dihydro-1*H*-pyrrole-3-carboxylate (2i)

Yield 0.48 g (58%), yellow crystals, m. p. 226-227°C (ethanol). IR spectrum, n, cm^{-1}: 3365, 3228, 3131, 1692, 1667, 1634, 1597. ^{1}H NMR spectrum (DMSO–d_6), δ, ppm: 1.26 t (3H, Me, J 7.1 Hz) , 4.21 q (2H, CH_2, J 7.1 Hz), 6.61 s (1H, CH), 6.62 double d (1H, furyl, J 3.5, 1.5), 7.09 double d (1H, furyl, J 3.5, 0.7), 7.24m (2 $N_{arom.}$), 7.80 m (2 $N_{arom.}$), 7.85 double d (1H, furyl, J 1.7, 0.7), 8.43 br. s (1H, NH), 9.15 br. s (1H, NH), 10.79 br. s (1H, NH). ^{13}C NMR spectrum (DMSO-d_6), d, ppm: 14.53, 58.55, 84.88, 102.19, 111.75, 115.57, 131.12, 134.01, 141.10, 145.83, 156.65, 167.01, 173.99, 189.91. Found, %: C, 58.14; H, 3.38; N, 10.14. $C_{20}H_{16}FN_3O_6$. Calculated, %: C, 58.11; H, 3.90; N, 10.17.

Ethyl 2-amino-5-(2-(4-chlorophenyl)-2-oxoethylidene)-1-(furan-2-carboxamido)-4-oxo-4,5-dihydro-1*H*-pyrrole-3-carboxylate (2j)

Yield 0.47 g (55%), yellow crystals, m. p. 255-256 °C (ethanol + dioxane). IR spectrum, n, cm^{-1}: 3413, 3127, 3109, 1707, 1656, 1628, 1610.

^{1}H NMR spectrum (DMSO–d_6), δ, ppm: 1.25 t (3H, Me, J 7.1 Hz), 4.19 q (2H, CH_2, J 7.1 Hz), 6.60 m (1H, CH, 1H, furyl), 7.07 m (1H, furyl), 7.23 d ($2H_{arom.}$J 7.9 Hz), 7.62 d ($2H_{arom.}$J 7.9 Hz), 7.82 m (1H, furyl), 8.36 br. s (1H, NH), 9.05 br. s (1H, NH), 10.68 br. s (1H, NH). Found, %: C, 55.85; H, 3.38; N, 9.81. $C_{20}H_{16}ClN_3O_6$. Calculated, %: C, 55.89; H, 3.75; N, 9.78.
